# On the Effects of Cinchona Bark, in Acute Gout and Rheumatism

**Published:** 1808-05

**Authors:** 


### *On the Effects of Cinchona Bark, in Acute Gout and Rheumatism. By Dr.* KINGLAKE.

AS the useful end of attentive observation is to ascertain what in the order of cause and effect is really true, and what erroneous, it may be presumed that some farther practical remarks on the efficacy of cinchona bark in acute gout and rheumatism, are worthy of being recorded in your Journal. The occasions for subjecting this efficient tonic to the test of experiment often present: the medical practitioner indeed, wishing to afford his pain-worn and exhausted patient the most direct and effectual relief, is almost irresistably invited to give the reputed remedy the earliest and most unwearied trial; at least, my own feelings have impelled me so to do, in instances sufficiently numerous, to enable me to form a decisive opinion on the subject.

It may be right to premise, that the true criteria of the acute state of arthritic or rheumatic affection are shewn by fullness and tension of pulse, severity of pain in the affected joints, urgent thirst, coated tongue, transient rigors succeeded by augmented heat, dry skin, convulsive startings, and sleepless nights. Where these symptoms exist, it is my accustomed practice to institute a cooling mode of treatment, even to the length of topically applying cold water to the inflamed joints. But this advice is not always strictly pursued, and is not unfrequently wholly resisted. Under these circumstances an attempt is made to reduce the prevailing inflammatory heat and action by freely evacuating the bowels, by moderate exposure to atmospheric cold, by copiously dilating with slender liquids, and by rigidly avoiding every kind of stimulant, whether of a medicinal or of a dietetic nature. The disease has commonly its original violence lessened by this mode of treatment; the pains soon remit, occasionally even intermit, the swellings diminish, and some advance is apparently made towards permanent recovery; but after the lapse of a few hours, rigor usually supervenes, followed either by an exasperated inflammation on the affected joints; or other joints, which had not previously suffered, become attacked.

This course of diseased action undermines the systematic strength, and entails on the afflicted patient an almost hopeless continuance of complaint. It is fair to infer from the representation that has been given of the efficacy of bark by Dr. Haygarth and others, that the conditions of disease disease here recited, are peculiarly those in which the remedy should exert its most curative influence; I have, however, to affirm, that the disease whilst retaining any evident appearance of an inflammatory state, has under my observation been uniformly aggravated by the medicine, and that even to such a degree as to render its discontinuance absolutely necessary. Additional pain and swelling of the affected joints, accelerated and hardened pulse, and strictured respiration have in various instances appeared to me to have directly resulted from employing the tonic agent.

Such an effect of the medicine, indeed, might, *a priori*, be reasonably expected from its well-known property of imparting additional tone and energy to the actions of life; but the importance of the subject, as well as the reputation of those who have authorised the practice, requires also the *a posteriori* proof, which correct experience alone can afford. No situation can be more harrassing than the usual lingering continuance of inflammatory affection of the joints. The highly irritated state of the system commonly obtaining in this disease often places the safety of the patient in no small risk. Nothing could be more gratifying, therefore, than to be able to render efficient aid in such embarrassing difficulties. Were the bark equal to that effect, its medicinal worth would be vastly enhanced; but unhappily for mankind, my experience obliges me to declare, that it appears in that state of disease to be decidedly, if not *necessarily*, injurious.

The advocates of this medicine, in cases of gouty and rheumatic inflammation, must have been deceived into a persuasion of its being generally useful in these instances, from finding it occasionally conducive to repairing the depredation on the systematic strength which an unchecked course of those diseases is apt to induce. There is certainly a period in the duration of inflammatory action at which that morbid state necessarily ends, owing to the vital energy requisite for the maintenance of such a condition of disease being exhausted; but before this spontaneous exit of inflammation happens, life itself might give way, or various kinds of incurable diseases proceeding from extreme debility might arise.

To rescue the drooping powers of life from the destructive exhaustion liable to be induced by an uncurtailed course of inflammatory disease, the bark would seem to be well adapted; and it is probable that its reputed salutary effects in arthritic and rheumatic affections may be justly ascribed ascribed to its tonic and renovating influence on such occasions. During the continuance of any degree of inflammatory swelling of the joints in arthritic and rheumatic cases, bark does not appear to be an appropriate medicine. Topical ablution of the affected joints with cold water, a well ventilated room, or, if practicable, moderate gestation, and a temperate course of diet, will be found to answer the purpose of promoting convalescence much more favourably than any description of medicinal stimulants.

Soda has likewise under my direction been subjected to extensive trial, as well in arthritic as in what are commonly considered as rheumatic affections, on the authority of Mr. Parkinson; but it does not appear to me that it has proved in any instance unequivocally beneficial. I do not know that it has ever been distinctly hurtful; it does not appear indeed to exert sufficient power to produce any positive effect as a medicine; in such cases, therefore, it would seem to be much too inert to justify any expectation of advantage from its employ.

The cooling treatment of gouty inflammation rests its claim to confidence on an enlarging basis of facts in its support. Since the recent publication of my "additional cases"* on the subject, fresh instances of the salutary effects of the practice have been made known to me, which, with many others, are in train for report, if farther intelligence in commendation of the treatment should be judged necessary.

*Taunton, March* 30, 1808.

---

### *To the Editors of the Medical and Physical Journal.*

Gentlemen,

As in the momentary quarrels of friends, the person who interferes, either impartially to conciliate, or particularly to assist, is seldom looked upon as a friend; or at least, is seldom thanked by either party: so, perhaps, in the disputes of your correspondents, it would be proper to leave the parties to decide them as they please. But as there

---

* See additional cases on the cooling treatment of gout, &c. By Robert Kinglake, M. D.

